# Phyllosphere mycobiome in two Lycopodiaceae plant species: unraveling potential HupA-producing fungi and fungal interactions

**DOI:** 10.3389/fpls.2025.1426540

**Published:** 2025-03-14

**Authors:** Liqun Lin, Cheng Li, Chiung-Chih Chang, Ran Du, Jiaojiao Ji, Li-Yaung Kuo, Ko-Hsuan Chen

**Affiliations:** ^1^ Shenzhen Branch, Guangdong Laboratory of Lingnan Modern Agriculture, Key Laboratory of Synthetic Biology, Ministry of Agriculture and Rural Affairs, Agricultural Genomics Institute at Shenzhen, Chinese Academy of Agricultural Sciences, Shenzhen, China; ^2^ Biodiversity Research Center, Academia Sinica, Taipei, Taiwan; ^3^ Institute of Molecular and Cellular Biology, National Tsing Hua University, Hsinchu, Taiwan

**Keywords:** huperzine A, mycobiome, fungal community, lycophyte, network analysis, co-cultivation, fungal facilitator

## Abstract

Huperzine A (HupA), a lycopodium alkaloid with therapeutic potential for neurodegenerative diseases such as Alzheimer’s disease, is found exclusively in some species of the Huperzioideae subfamily of Lycopodiaceae. Fungi associated with Huperzioideae species are potential contributors to HupA biosynthesis, offering promising prospects for HupA production. Despite its medical significance, limited knowledge of fungal diversity in lycophytes and the variability of HupA production in fungal strains have impeded the discovery and applications of HupA-producing fungi. Here, we investigated HupA concentrations and the mycobiome across various tissues of two Lycopodiaceae species, *Huperzia asiatica* (a HupA producer) and *Diphasiastrum complanatum* (a non-HupA producer). We aim to unveil the distribution of potential HupA-producing fungi in different plant tissues and elucidate fungal interactions within the mycobiome, aiming to uncover the role of HupA-producing fungi and pinpoint their potential fungal facilitators. Among the tissues, *H. asiatica* exhibited the highest HupA concentration in apical shoots (360.27 μg/g fresh weight) whereas *D. complanatum* showed no HupA presence in any tissue. We obtained 441 amplicon sequence variants (ASVs) from *H. asiatica* and 497 ASVs from *D. complanatum*. The fungal communities in bulbils and apical shoots of *H. asiatica* were low in diversity and dominated by Sordariomycetes, a fungal class harboring the majority of reported HupA-producing fungi. Integrating bioinformatics with published experimental reports, we identified 27 potential HupA-producing fungal ASVs, primarily in *H. asiatica*, with 12 ASVs identified as hubs in the fungal interaction network, underscoring their pivotal roles in mycobiome stability. Members of certain fungal genera, such as *Penicillium*, *Trichoderma*, *Dioszegia*, *Exobasidium*, *Lycoperdon*, and *Cladosporium*, exhibited strong connections with the potential HupA producers in *H. asiatica*’s network rather than in *D. complanatum*’s. This study advances our knowledge of fungal diversity in Lycopodiaceae and provides insights into the search for potential HupA-producing fungi and fungal facilitators. It highlights the importance of exploring young tissues and emphasizes the ecological interactions that may promote the fungi-mediated production of complex bioactive compounds, offering new directions for research in fungal ecology and secondary metabolite production.

## 1 Introduction

The phyllosphere mycobiome refers to fungal communities that inhabit both the surface (epiphytes) and interior (endophytes) of aboveground plant tissues (Dong et al., 2021). While some phyllosphere fungi are known for their ability to promote plant growth and health, the functions of most of them remain understudied (Rodriguez et al., 2009). Recently, endophytic fungi have garnered attention as potential sources of bioactive compounds, especially in medicinal plants that harbor a wide range of endophytes (Venieraki et al., 2017; Gupta et al., 2020; Tiwari and Bae, 2022). Research has shown that some endophytic fungi can exert beneficial effects on host metabolism by producing, inducing, or modifying plant-derived natural products (Kusari et al., 2012). Biosynthesis of several well-known plant-derived bioactive compounds facilitated by endophytic fungi include taxol, camptothecin, podophyllotoxin, and vincristine (Chandra, 2012; Perez-Matas et al., 2023). These findings provide a promising avenue for developing an environmentally friendly, relatively simple, and cost-effective alternative method for producing bioactive compounds (Venieraki et al., 2017; Daley and Cordell, 2021; Li et al., 2022).

Huperzine A (HupA) is a natural lycopodium alkaloid isolated from the traditional Chinese medicine (TCM) “QianCengTa” (Wang et al., 2006). This compound has received significant attention as an anti-Alzheimer’s disease drug candidate, a condition which is the leading cause of dementia in older adults, which afflicts millions worldwide (Ren et al., 2022). HupA has also shown promise in other neurodegenerative diseases and aging-related treatments (Mukherjee et al., 2007; Shen et al., 2017). The increase of ageing populations is leading to an escalating demand for HupA, resulting in a supply–demand imbalance. Currently, HupA is primarily obtained through natural extraction and chemical synthesis. Certain species of the Huperzioideae subfamily in Lycopodiaceae are the only known natural source of HupA, whereas the sister lineages, Lycopodioideae and Lycopodielloideae (**Supplementary Figure S1**), do not produce HupA (Halldorsdottir et al., 2015; Zhu and Zhao, 2019). Extracting HupA from Huperzioideae species faces several challenges, including limited abundance, long growth cycle, low HupA content, and difficulties in cultivation (Ma et al., 2007). Moreover, environmental deterioration and overexploitation of wild *Huperzia serrata* (the plant source of TCM “QianCengTa”) populations have raised apprehensions about the sustainability of HupA supply (Chen et al., 2021). Chemical synthesis of HupA is also limited due to its complex structure, low productivity, and the fact that the synthesized *(+)-HupA* isomer is less effective than the naturally occurring *(−)-HupA* isomer, particularly in inhibiting acetylcholinesterase (Yang et al., 2020). Moreover, synthetic production is typically more costly compared with plant extraction, further complicating large-scale production.

Recent research has highlighted the potential of endophytic fungi in HupA production, offering a promising alternative to the challenges associated with natural extraction and chemical synthesis. The mycobiome of Huperzioideae species may play a key role in HupA production, with some fungal species potentially inducing HupA accumulation in the host plants (Cao et al., 2021; Cui et al., 2021; Shen et al., 2022). Various endophytic fungi have been found to produce HupA independently in culture, without the need for cooperation with the host plant (**Supplementary Table S1**). These fungi offer a sustainable avenue of HupA production without large-scale cultivation or harvesting of the host plant. Their growth and metabolism can also be manipulated to increase the yield of HupA, making these fungi commercially valuable (Sang et al., 2020).

However, as of yet, none of the identified HupA-producing fungi have successfully been employed for HupA industrial production. Lycopodiaceae plays a significant ecological role in various ecosystems, particularly through its mycorrhizal associations, including fine-root endophytes (George and Bazzaz, 1999; Kowal et al., 2020, Kowal et al., 2022; Perez-Lamarque et al., 2023). However, research on the foliar mycobiome of Lycopodiaceae has primarily been focused on a few well-known HupA-producing species, resulting in a limited understanding of fungal diversity within the broader range of Lycopodiaceae species, thus impeding the discovery of new fungal species with high HupA production capacity (Fan et al., 2020; Shen et al., 2022). Another major hurdle is that the repeated subculturing of endophytic fungi leads to diminished or complete shutdown of HupA production over generations (Deshmukh et al., 2019; Deepika et al., 2020), which could partially be attributed to the absence of other interactive fungi inhabiting the same ecological niche. The current process of identifying potential fungi producing specific secondary metabolites revolves around isolation, purification, and subculturing, often disregarding the probable impact of endophytic fungal interactions on secondary metabolite production (Venugopalan and Srivastava, 2015). Additionally, most strains isolated from the host plant are discarded without further exploration (Kusari and Spiteller, 2012). Nevertheless, several studies have demonstrated that co-cultivation of different fungi sharing the same ecological niche can significantly enhance the production of secondary metabolites in target fungi (Soliman and Raizada, 2013; Bhalkar et al., 2016). However, co-cultivation applications for the production of fungal HupA have not been reported so far. Thus, investigating the interactions among fungi associated with Huperzioideae species, particularly those that produce HupA, could provide valuable insights into the correlation between metabolite content and fungal interaction networks.

In this study, a high-throughput sequencing method was employed to investigate the diversity and composition of endophytic fungal communities in various tissue parts of two lycophytes, *Huperzia asiatica* (producing HupA) and *Diphasiastrum complanatum* (not producing HupA). We utilized two approaches, sequence clustering and correlation analysis, to identify the potential HupA-producing fungi in *H. asiatica*. Furthermore, we employed networks analysis to reveal fungal interactions within the mycobiome. We aim to 1) characterize the mycobiome assemblages associated with *H. asiatica*, the HupA-producing plant; 2) assess the significance of potential HupA-producing fungi within the mycobiome networks of HupA-producing and non-HupA-producing lycophytes; and 3) identify potential facilitators that exhibit close interactions with HupA-producing fungi. Overall, our goal is to advance our understanding of the intricate interactions between phyllosphere fungi and natural product biosynthesis, and thus to promote the application of fungi in the industrial production of plant-derived bioactive compounds.

## 2 Materials and methods

### 2.1 Plant materials

**Figure 1:**
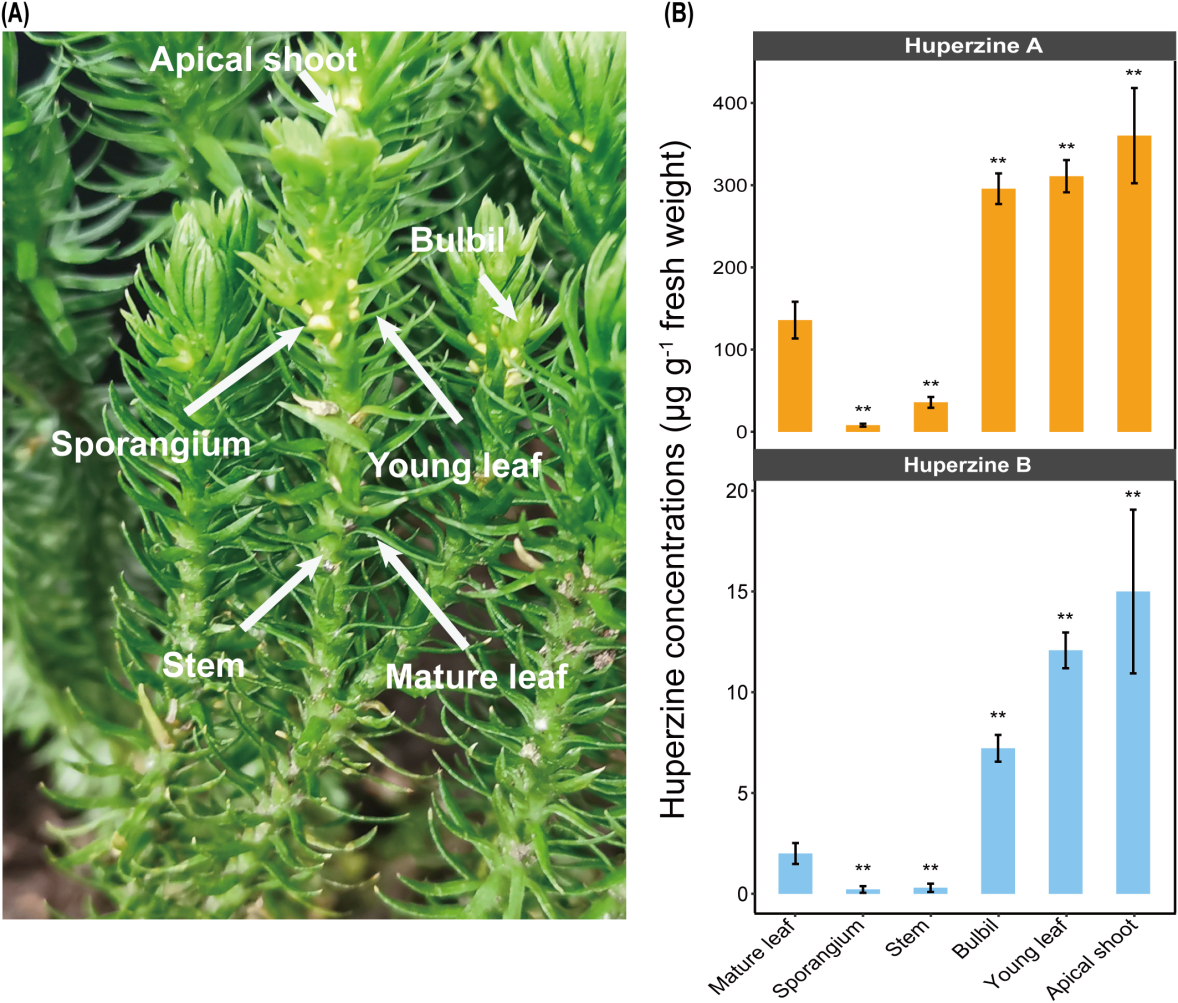
Huperzine concentrations. **(A)** Tissues of *H. asiatica* plants (apical shoot, bulbil, young leaf, mature leaf, stem, and sporangium) are used to quantify huperzine contents and to extract DNA for subsequent amplicon sequencing analysis. **(B)** Huperzine concentrations (micrograms per gram fresh weight) in diverse tissues (Wilcoxon signed rank test, **P < 0.01). Initial concentration data are provided in **Supplementary Table S2**.

The HupA-producing plants *H. asiatica* were collected in September 2019 from Changbai Mountain National Nature Reserve in Antu County, Jilin Province, China (41°41′49"-42°25′18"N, 127°42′55"-128°16′48"E, 2,000-3,000 m above sea level). The non-HupA-producing plants *D. complanatum* were collected in June 2020 from Taipingshan National Forest Recreation Area in Yilan County (24° 30′ 10" N, 121° 31′ 55" E, 2,000 m above sea level). We sampled leaves and shoots from both species (**Figure 1A**; **Supplementary Figure S2**), as HupA has been identified in its highest concentrations within these aboveground vegetative organs (Shen et al., 2022). For *H. asiatica*, we further divided the leaf part into newly generated (young leaf) and fully expanded ones (normal leaf) that were located distantly from the apices (**Figure 1A**). Additionally, the reproductive organs, including sporangia and bulbils, were sampled in *H. asiatica*. This finer sampling approach allows us to accurately identify the specific tissue responsible for the HupA production in *Huperzia*. To ensure consistency, three biological replicates from each tissue type were collected for liquid chromatography-mass spectrometry (LC-MS) detection of Huperzines and six replicates were collected for fungal community detection using amplicon sequencing.

### 2.2 LC-MS detection of huperzines

All common chemicals and reagents were obtained from commercial vendors. Huperzine standards were purchased from commercial vendors: Huperzine A (APExBIO Technology LLC, Houston, TX, USA) and Huperzine B (MilliporeSigma, Burlington, MA, USA).

To measure the huperzine concentrations in plant tissues, samples were ground to fine powder in liquid nitrogen and weighed to calculate the fresh mass. Prepared samples were analyzed with an AB SCIEX TripleTOF 6600 Plus system (AB SCIEX, MA, USA). 2 µL of extracts was injected and separated by an AB SCIEX ExionLC system (AB SCIEX, MA, USA) equipped with an ACQUITY UPLC™ BEH C18 column (Waters Ltd., MA, USA) (150 mm × 2.1 mm, 2.6 μm). Water with 0.1% formic acid was used as mobile phase A, and ACN with 0.1% formic acid was used as mobile phase B, and the flow rate was 0.2 mL/min. A 20-min gradient method was used with mobile phase B gradually increased from 3% to 97%. Electrospray ionization (ESI) positive mode was used for detection, and the source conditions were set as follows: Ion Source Gas1 (Gas1) of 60, Ion Source Gas2 (Gas2) of 60, curtain gas (CUR) of 30, source temperature of 600°C, and IonSpray Voltage Floating (ISVF) 5,500 V. Data with mass ranges of m/z 120-1,800 were acquired under data-dependent MSMS acquisition mode. In auto MS/MS acquisition, the instrument was set to acquire over the m/z range 25-1,000 Da. The product ion scan parameters were set as follows: the collision energy (CE) was fixed at 35 V with ± 15 eV, and the declustering potential (DP) was 60 V (+). Data were then analyzed by a SCIEX OS software (version 1.7, AB SCIEX, MA, USA). The presence of HupA and B in these samples was confirmed via both retention time and MS/MS data, and the concentration was calculated by calibration curves made with standards (**Supplementary Figure S3**). Huperzine concentrations in *H. asiatica* across different tissues are shown in **Supplementary Table S2**.

### 2.3 Amplicon sequencing

After collection, the samples were stored *in silica* gels until DNA extraction. We used a different number of pieces per DNA extraction for different tissue types due to the differences in their sizes (approximately one 2.5-cm stem of *D. complanatum* and *H. asiatica*, one 2.5-cm leaf of *D. complanatum*, five 0.5-cm bulbil/apical shoots of *H. asiatica*, and 20 0.3-cm young leaf/mature leaves of *H. asiatica* per DNA extraction). We ground the plant tissues in a 2.0-mL screw tube with one 3.2-mm-diameter stainless steel bead and two 2.3-mm-diameter zirconia/silica beads (BioSpec Products, Bartlesville, Oklahoma, USA). The tissues were ground at 60 Hz for 30 s three times using a STEP tissue grinder (ACTTR, New Taipei City, Taiwan). The DNA sample was extracted following the protocol of the NautiaZ Plant DNA Mini Kit (Nautia Gene, Taipei, Taiwan) and eluted with 35 µl UltraPure™ DNase/RNase-Free Distilled Water (Invitrogen, Carlsbad, CA, USA). We used a two-step PCR approach with the ITS3ngs and ITS4 primers following Chen et al. (2021) (**Supplementary Table S3**) to amplify the nuclear intergenic spacer 2 of the ribosomal DNA (nrITS2). The PCR product was examined on gel electrophoresis and cleaned up by the AMPure XP beads (PCR product: bead 1:1 v:v, Beckman Coulter, Krefeld, Germany). The generated libraries were pooled with equal molar DNA, predetermined by Qubit HS dsDNA HS Assay Kit (Invitrogen, Carlsbad, CA, USA), and sequenced with the Illumina MiSeq platform (300-bp paired ends) at the NGS High Throughput Genomics Core at Academia Sinica, Taipei. The read data are available in the Short Read Archive (SRA) of NCBI GenBank (BioProject: PRJNA981640).

### 2.4 Data processing

Raw reads were demultiplexed and the primers were removed by cutadapt (Martin, 2011). Next, DADA2 (Callahan et al., 2016) was applied to filter out low-quality reads, merge paired-end reads, define unique sequences known as amplicon sequence variants (ASVs), and eliminate chimeric sequences. Subsequently, LULU (Frøslev et al., 2017) was utilized for post-clustering of ASVs, removing artificial or intraspecific ASVs that could have arisen from sequencing errors or PCR artifacts. This resulted in a refined ASV table. The taxonomic identity of ASVs was assigned via the RDP Naive Bayesian Classifier algorithm implemented in DADA2, utilizing the UNITE eukaryote database (Nilsson et al., 2019). Finally, the R package “phyloseq” (McMurdie and Holmes, 2013) was used to compute alpha diversity indices (observed species, Shannon index) and beta diversity using the Bray–Curtis dissimilarity measure. A species accumulation curve was examined with the R package ggrare (https://rdrr.io/github/gauravsk/ranacapa/man/ggrare.html) to ensure sequencing adequacy (**Supplementary Figure S4**).

### 2.5 Identification of potential HupA-producing fungi

We determined the potential HupA-producing fungal ASVs with two approaches: 1) sequence clustering and 2) correlation analyses between HupA content and mycobiome abundance. To gather primary literature on endophytic fungi capable of producing HupA, we conducted a comprehensive literature search in the China National Knowledge Infrastructure (CNKI) database (https://www.cnki.net/) and the Institute of Scientific Information’s Web of Science (WOS) database (https://www.webofscience.com/wos). We focused only on studies that were verified through culture experiments. As of October 20, 2022, we compiled a total of 36 articles reporting HupA-producing fungal strains, comprising 48 strains in total (**Supplementary Table S1**). We then clustered our ASV sequences with these sequences of known HupA-producing strains by VSEARCH (Rognes et al., 2016) and Geneious Prime 2022.1.1 (https://www.geneious.com) at 97% similarity. As for the correlation analyses, given that the HupA concentration and mycobiomes were not derived from the same plant, we used the mean HupA concentration of three replicates per tissue type to conduct the correlation test (Kendall’s tau rank sum correlation).

### 2.6 Statistical analysis

To compare the fungal community assemblies in different tissues, we conducted permutational multivariate analysis of variance (PERMANOVA) test with 999 permutations. To test for differences in alpha diversity across tissue types, we conducted ANOVA test with *post hoc* Tukey HSD test. If the data do not follow a normal distribution, a Kruskal–Wallis test followed by Dunn test was applied instead. The P value was corrected by Holm–Bonferroni for multiple comparison corrections. To compare the diversity between the two plant species, a Wilcox rank-sum test was performed. For analysis that required data normalization, we normalized the raw read number to percentage per sample. For alpha diversity measurements, we rarefied the data to 26,328 reads, the lowest number of reads in a sample.

### 2.7 Nestedness analysis of mycobiome

To investigate mycobiome nestedness, we analyzed the distribution of fungi in different plant tissues of *H. asiatica*. Specifically, the ASVs table was merged by sample tissues, resulting in a presence/absence matrix, in which each row represented a plant part and each column indicated an ASV. A value of “1” indicated the presence of an ASV in a particular plant part, whereas a “0” indicated its absence. We employed the NODF (Nestedness based on Overlap and Decreasing Fill) metric to quantify the degree of nestedness, ranging from 0 (no nesting) to 100 (perfect nesting) (Almeida-Neto et al., 2008). Additionally, we calculated the nestedness temperature (Atmar and Patterson, 1993), which reflects the degree of nestedness, with lower values indicating a more nested community structure. To validate the accuracy of our nestedness model, we used null models that only preserves the number of presences of ASVs in the matrix while randomizing the distribution across tissues. We performed the data analysis using R packages “bipartite” (Dormann et al., 2008) and “vegan” (Oksanen et al., 2007). To assess the statistical significance of nestedness temperature in our dataset, we conducted 1,000 null model simulations.

### 2.8 Mycobiome comparison between *H. asiatica* and *D. complanatum*

To compare the fungal communities between the two species, we combined the two leaf subtypes collected for *H. asiatica* (mature leaf and young leaf) as we did not separate the leaves into subtypes for *D. complanatum*. We then investigated the alpha and beta diversity between the two species, as described above. To identify differentially abundant ASVs between *H. asiatica* and *D. complanatum*, we used R packages “DESeq2” (Love et al., 2014), which utilizes a negative binomial generalized linear model of reads. As the leaves of *H. asiatica* had a much higher HupA concentration compared with its stems, we focused the differentially abundant test on ASVs of leaves.

### 2.9 Network analyses

To further reveal ASV interactions, network analyses were carried out. The datasets were split into three categories: A. HupA-rich tissues of *H. asiatica* (including apical shoots, bulbils, and young leaves), B. leaves and stems of *H. asiatica*, and C. leaves and stems of *D. complanatum*. ASVs with more than 25 sequences of each category were selected to calculate possible correlations. FlashWeave version 0.19.1, a novel co-occurrence method that predicts microbial interaction networks through graphical model inference (Tackmann et al., 2019), was used to construct fungal interaction networks, using default parameters. Network properties were calculated in R version 4.2.2. The fast greedy algorithm was used to detect communities (Deng et al., 2012), based on which within-module connectivity (Zi) and among-module connectivity (Pi) were calculated to statistically identify microbial keystone ASVs. Visualization of the constructed networks was performed by Gephi version 0.9.2 (Bastian et al., 2009).

The distribution curves of network node degrees were analyzed to assess their fit with the power-law model using R² as the goodness-of-fit metric (R² > 0.9; **Supplementary Figure S6**). To assess non-random patterns in the resulting network, we compared our networks against the random networks with equal nodes and edges. The cohesive characteristics of the network, including modularity and average clustering coefficient, were calculated and compared with those of a corresponding random network to evaluate the presence of non-random interactions among mycobiome species. These analyses were conducted to confirm whether these networks conform to the general rule of microbial interaction networks and can be used for subsequent statistical analyses (Zhou et al., 2010; Deng et al., 2016; Yang et al., 2021).

## 3 Results

### 3.1 Huperzines in tissues of *H. asiatica*

We examined the levels of HupA and its analog HupB, which is considered as a precursor of HupA (Nett et al., 2021), in different tissues of *H. asiatica* (**Figure 1A**). In general, both huperzines showed a similar accumulation pattern in the different tissues of *H. asiatica*: younger tissues had higher huperzine concentrations (Wilcoxon signed rank test: *P* < 0.01; **Figure 1B**). For example, the apical shoots of *H. asiatica* had the highest huperzine concentrations (360.27 μg/g fresh weight for HupA and 15.00 μg/g fresh weight for HupB, respectively), whereas the mature leaves exhibited the lowest huperzine concentrations (135.90 μg/g fresh weight for HupA and 2.00 μg/g fresh weight for HupB, respectively). In addition, we found that huperzines were not detected in any tissue of another lycophyte from the Lycopodiaceae, *D. complanatum*, which is consistent with previous reports (Halldorsdottir et al., 2015). These results suggested that *H. asiatica* and *D. complanatum* provided a contrast system to study mycobiome related to HupA biosynthesis.

### 3.2 Sequencing results

After quality filtering of reads and removal of non-fungal taxa, we obtained 74,231 ± 25,233 fungal reads for each sample. A total of 925 ASVs were detected (**Supplementary Table S4**). In *H. asiatica* samples, 441 ASVs were discovered, including 217 in stems, 184 in mature leaves, 183 in young leaves, 97 in sporangia, 56 in bulbils, and 55 in apical shoots (**Supplementary Figure S5**). In *D. complanatum* samples, 497 ASVs were retrieved, including 319 in leaves and 273 in stems (**Supplementary Figure S5**). Only 13 ASVs were commonly shared between the two species.

### 3.3 Assembly of a list of potential HupA-producing fungi

We further collected the reported HupA-producing fungal strains and their ITS sequences from published literatures. A table containing 48 strains was assembled, most of which belonged to the class Sordariomycetes (**Supplementary Table S1**). At the genera level, *Colletotrichum* (n = 9), *Fusarium* (n = 6), and *Penicillium* (n = 6) are the most dominant genera. A total of 20 genera, in which these fungi were identified, were subsequently referred to as reported HupA-producing (RHP) genera for brevity. By sequence clustering, a total of 24 ASV sequences passing the 97% sequence similarity cutoff clustered with the reported HupA-producing fungi. Of these 24 ASVs, 18 were identified by both VSEARCH and Geneious, whereas six were identified only by Geneious (**Supplementary Table S5**). Furthermore, we performed the correlation analyses between the relative abundance of ASVs and the concentration of HupA across *H. asiatica*’s tissues, by which four ASVs with correlation coefficient (Kendall’s tau b) > 0.4 were identified (**Supplementary Table S5**). One ASV (ASV10, *Penicillium citrinum*) was identified by both the sequence clustering and correlation analyses. The two analyses rendered a total of 27 ASVs, referred to as potential HupA-producing (PHP) ASVs for subsequent analyses.

### 3.4 Mycobiome among tissues of *H. asiatica*

**Figure 2:**
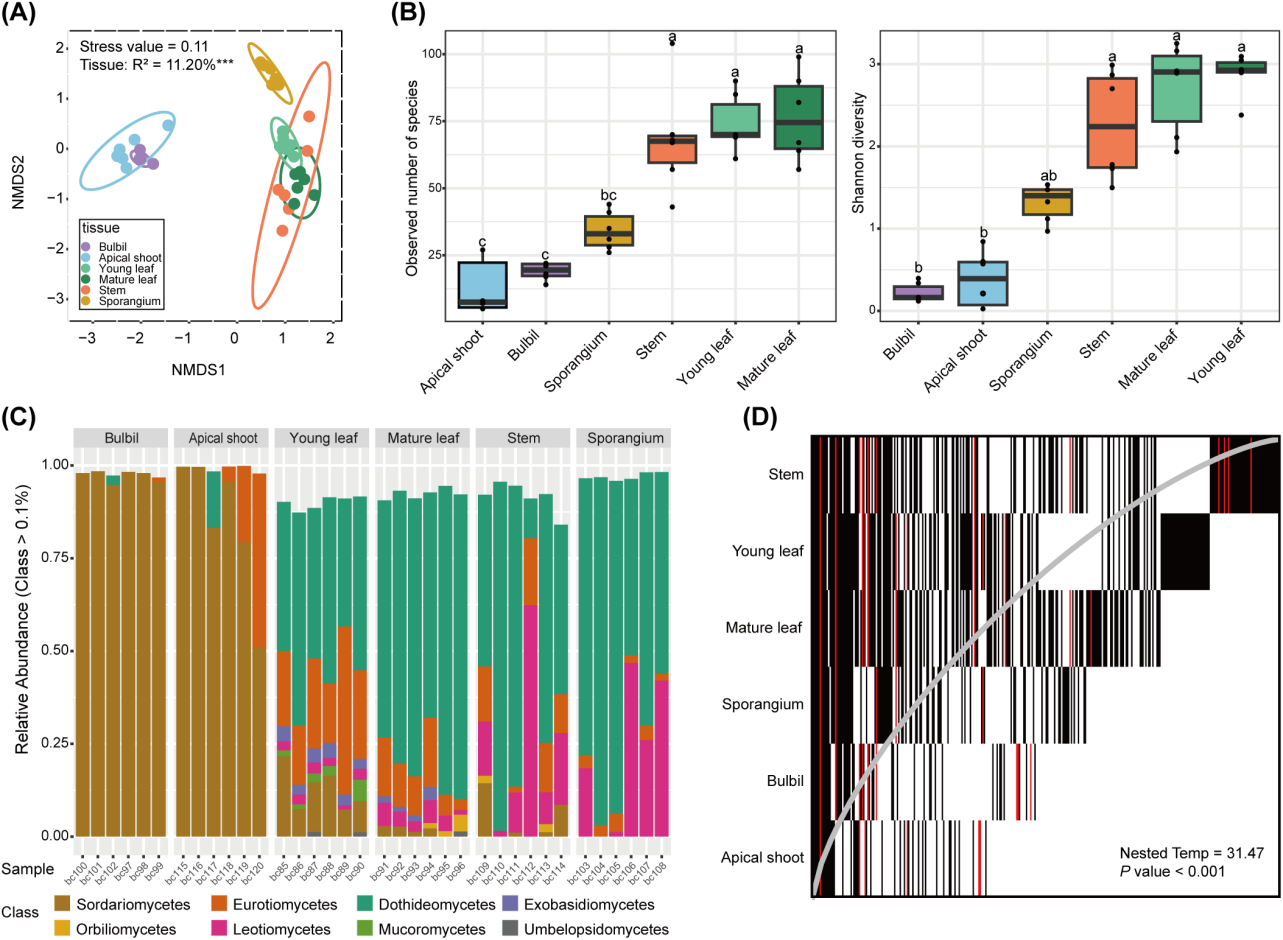
Mycobiome features of *H. asiatica*. **(A)** Non-metric multidimensional scaling (NMDS) ordination plot based on the Bray–Curtis dissimilarity of (*H*) *asiatica* mycobiomes. ***P value < 0.001. Kruskal’s stress values are presented (values less than 0.2 represent good ordination plots). **(B)** Alpha diversity across tissue types in *H. asiatica*. Significant groups in pair-wise comparisons (*p*-adjusted value < 0.05) are indicated by different letters above the boxplots. **(C)** Mycobiome compositions of *H. asiatica* at the class level. The horizontal axis represents the sample name and the vertical axis shows the relative proportion of ASVs annotated to a certain class. The class categories corresponding to each color block are shown in the legend on the right. **(D)** Nestedness plot of fungi aggregated by plant tissues. Each rectangle represents the presence of an ASV in a particular plant tissue. ASVs are arranged from left to right based on their occupancy across plant tissues, and the rows are ordered by decreasing ASV richness from top to bottom. The gray line represents the isocline, and a perfectly nested matrix would have all ASVs on the left side of the isocline.

We next examined the fungal community in different tissues of *H. asiatica*. Mycobiome structures of *H. asiatica* are significantly different across tissue types (*P* = 0.001; **Figure 2A**). The mycobiomes of bulbils and apical shoots are similar to each other while distinct from young leaves, mature leaves, stems, and sporangia (**Figure 2A**). Alpha diversity is significantly different across tissue types (*P* < 0.001), with bulbils and apical shoots having the lowest fungal diversity (**Figure 2B**). At the fungal class level, bulbils and apical shoots are dominated by Sordariomycetes, whereas the other tissues have high proportions of Dothideomycetes (**Figure 2C**). Based on our PHP list, both Sordariomycetes and Dothideomycetes have been reported to produce HupA. The nestedness temperature of *H. asiatica*’s mycobiomes is 31.478, which is significantly lower than the simulated one (*P* < 0.001), suggesting that the mycobiomes exhibit some degree of nestedness. Stems contain the majority of mycobiomes detected in other tissues (**Figure 2D**). Additionally, the matrix fill value is 0.342, which is the mean of non-diagonal elements, indicating that there are relatively few shared ASVs among tissues. The distribution of PHP ASVs (red) is consistent with the overall nestedness structure (**Figure 2D**). Stem contains most species of PHP ASVs, whereas the phyllosphere (apical shoot, bulbil, and leaves) contains some unique PHP ASVs, suggesting that PHP ASVs may originate from different sources.

### 3.5 Mycobiome comparison between *H. asiatica* and *D. complanatum*

**Figure 3:**
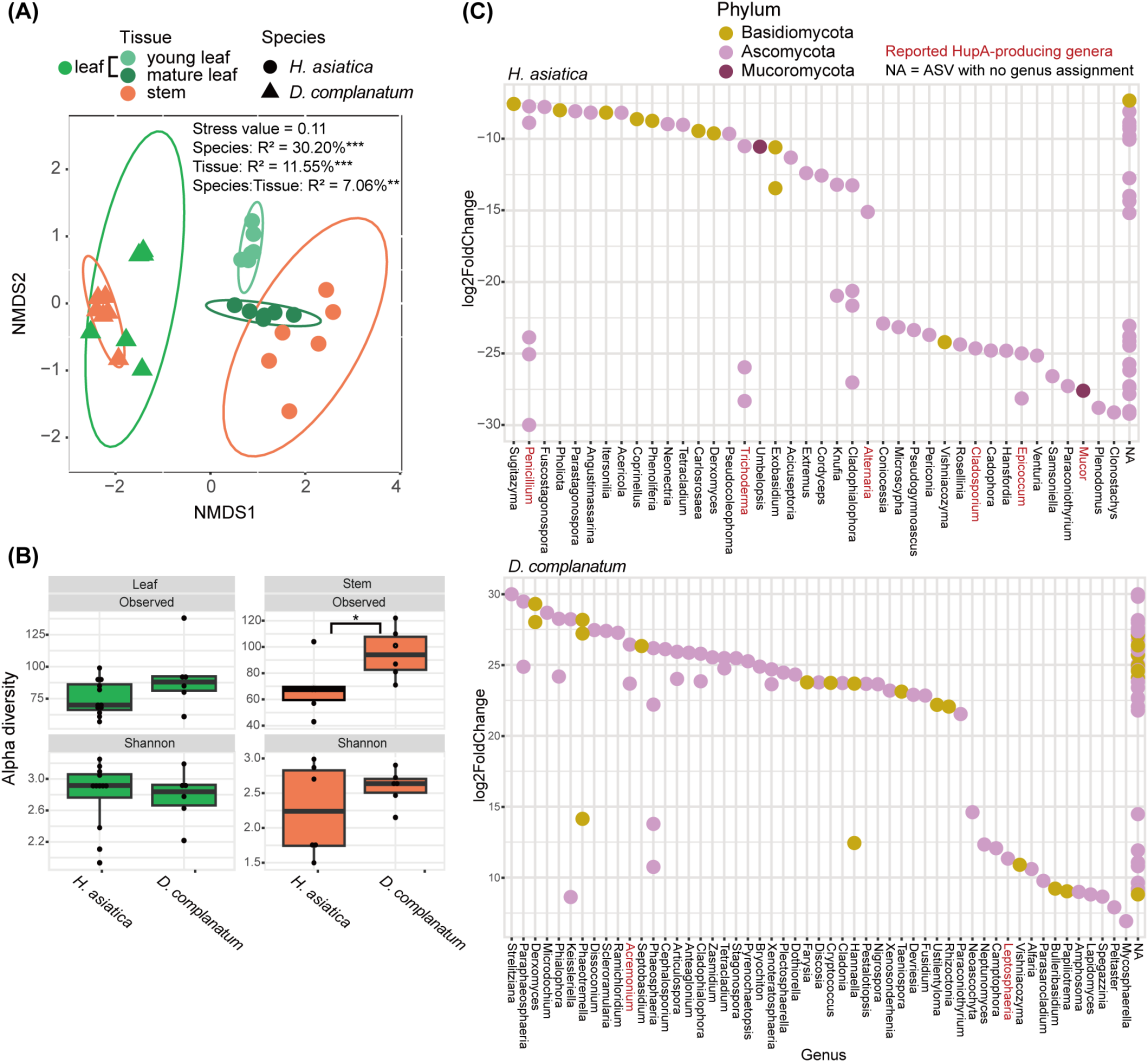
Comparison of mycobiome in the leaves and stems of *H. asiatica* and *D. complanatum*. **(A)** Non-metric multidimensional scaling (NMDS) ordination plot based on the Bray–Curtis dissimilarity of *H. asiatica* and *D. complanatum* mycobiomes. **(B)** Alpha diversity of endophytic fungi in leaves and stems of *H. asiatica* and *D. complanatum*. **(C)** Differentially abundant taxa between the leaves of *H. asiatica* and *D. complanatum* (Wald test, *padj* < 0.01, log_2_FoldChange > 2). The red font denotes genus with HupA-producing potential in the literature. ***P value < 0.001, **P value < 0.01, *P value < 0.05.

The fungal communities of leaf and stem were further compared between the HupA-producing plant *H. asiatica* and the non-HupA producing plant *D. complanatum*. The community assemblies of leaf and stem were significantly different between the two species and two tissue types (*P* < 0.01, **Figure 3A**). The number of observed mycobiome species in stems were significantly higher in *D. complanatum* than that in *H. asiatica* (Kruskal–Wallis test, *P* = 0.02, **Figure 3B**). No differences were detected for Shannon diversity in the two species (**Figure 3B**). We then identified the differentially abundant ASVs between the leaves of *H. asiatic* and *D. complanatum*. A total of 71 and 111 ASVs were significantly (*padj* < 0.01, log_2_FoldChange >2) more abundant in *H. asiatica* and *D. complanatum*, respectively. Of the 71 abundant ASVs in *H. asiatica* leaves, seven were identified as PHP ASVs. In contrast, only three out of 111 abundant ASVs in *D. complanatum* were the PHP ASVs. By investigating the genus assignments of the significantly differentially abundant ASVs, six (*Penicillium*, *Trichoderma*, *Alternaria*, *Cladosporium*, *Epicoccum*, and *Mucor*, comprising a total of 13 ASVs) and two genera (*Acremonium* and *Leptosphaeria*, comprising a total of 3 ASVs) with reported HupA-producing ability were detected in *H. asiatica* and *D. complanatum*, respectively (**Figure 3C**).

### 3.6 Network analyses revealed potential HupA-producing ASVs’ interactions

**Figure 4:**
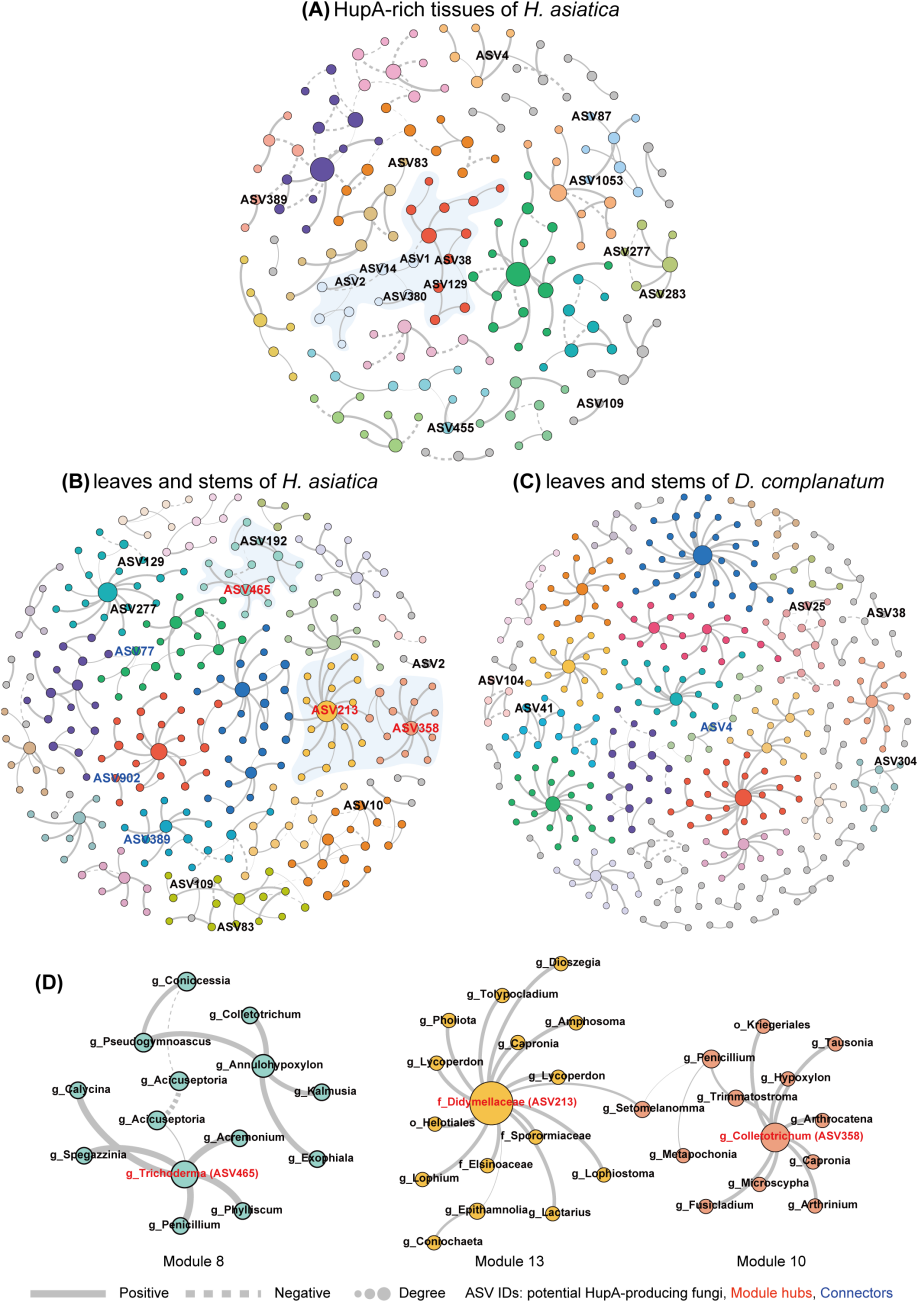
Co-occurrence networks of *H. asiatica* and *D. complanatum* mycobiome community. Networks of **(A)** HupA-rich tissues of *H. asiatica* (including apical shoots, bulbils, and young leaves); **(B)** leaves and stems of *H. asiatica*; and **(C)** leaves and stems of *D. complanatum*. The networks are visualized based on modules. Large modules with ≥5 nodes are shown in different colors, and smaller modules are shown in gray. Nodes represent different ASVs, and the size of each node is proportional to the number of degrees. Edges refer to the correlation among nodes, being either positive (solid) or negative (dashed). ASV IDs of potential HupA-producing fungi were labeled. **(D)** The modules containing potential HupA-producing fungal ASVs as hubs in the network of leaves and stems in *H. asiatica*. Details of network topological attributes are listed in **Supplementary Table S6**. The node information for the three networks is in **Supplementary Tables S7**–**S9** respectively.

We further constructed three interaction networks (A. HupA-rich tissues of *H. asiatica*, B. leaves and stems of *H. asiatica*, and C. leaves and stems of *D. complanatum*) to model and simplify the complexity of microbial interactions, offering insights beyond traditional abundance-based analyses. **Supplementary Table S6** shows the main topological properties of the three networks. We visualized network A based on modules and labeled the PHP ASVs. The network included 28 modules, nine of which contain more than 4% nodes (**Supplementary Table S7**). Interestingly, some of these PHP ASVs clustered together. For example, ASV1, ASV2, ASV14, and ASV380 were embedded in module 5 and were connected to module 3 containing ASV38 and ASV129 (**Figure 4A**). Both network B (leaves and stems of *H. asiatica*) and network C (leaves and stems of *D. complanatum*) show high complexity and modularity. However, only five nodes and no edges are shared between the two networks, suggesting that the two plant species have completely different fungi despite that we sampled the same tissue. Consistent with our expectations, *H. asiatica*’s network contained more PHP ASVs than *D. complanatum*’s (13 and 6, respectively).

We identified the different roles of the nodes in the three networks based on intra-module connectivity (Zi) and inter-module connectivity (Pi) plots (**Supplementary Figure S7**). *H. asiatica*’s network (network B) has 12 module hubs (including three PHP ASVs) and 70 connectors (including three PHP ASVs) (**Supplementary Table S8**; **Figures 4B, D**), whereas *D. complanatum*’s network (network C) has 10 module hubs and 59 connectors (including one PHP ASVs) (**Supplementary Table S9**; **Figure 4C**). Furthermore, we examined the main fungal genera within modules containing PHP fungi in each of the three networks (**Supplementary Figure S8**). The composition of these genera differed between the networks of the two species (*H. asiatica* and *D. complanatum*). Specifically, in both networks A and B of *H. asiatica*, ASVs of the genera *Penicillium*, *Trichoderma*, *Dioszegia*, and *Exobasidium* were detected in multiple modules containing PHP fungi. In contrast, in *D. complanatum*’s network (network C), members of the family Didymellaceae and the genera *Phaeosphaeria* exhibited more connections with PHP fungi. These findings highlight the distinct composition of fungal genera associated with PHP fungi between the two species.

## 4 Discussion

The discovery of new fungal species with HupA-producing capabilities involves a series of necessary while laborious processes (Venugopalan and Srivastava, 2015). Several practical bottlenecks, such as low yield and attenuation of metabolite production during *in vitro* subculturing, limit the commercial success of HupA-producing fungi (Deshmukh et al., 2019). This study represents the first comprehensive analysis of phyllosphere mycobiomes in *H. asiatica* and *D. complanatum*, two Lycopodiaceae plants with and without capacities to produce HupA, respectively. Moreover, our study offers strategies to increase the likelihood of identifying fungi that could be applied to, or could facilitate, the large-scale commercial production of HupA.

Our study reveals a correlation between HupA content and the diversity distribution patterns of fungi across tissues of *H. asiatica*, consistent with previous studies on *H. serrata* (Shen et al., 2022). Notably, we observed a distinct pattern in young tissues, such as bulbils and apical shoots, where the fungal diversity was the lowest despite the highest accumulation of HupA. We postulate that young tissues have limited time for fungal colonization, while increased light exposure due to their positioning on the plant contributes to the high content of HupA (Guo et al., 2018; Gupta et al., 2020). Despite of the low fungal diversity in bulbils, the proportion of potential HupA-producing ASVs is the highest in this tissue (16.07% in bulbils, 10.91% in apical shoot, 5.46% in young leaf, 5.42% in mature leaf, 5.52% in stem, and 5.15% in sporangium). The evidence makes bulbils a promising source for the isolation of HupA-producing fungi, in addition to its function as vegetative propagules (Simpson, 2019). In short, our study raises new candidate tissues for isolating HupA-producing fungi, besides the previously proposed tissues of leaves and stems (Cui et al., 2021), which encompass the majority of identified HupA-producing strains (**Supplementary Table S1**) but also comprise other fungal strains enlarging the difficulties of isolation.

**Figure 5:**
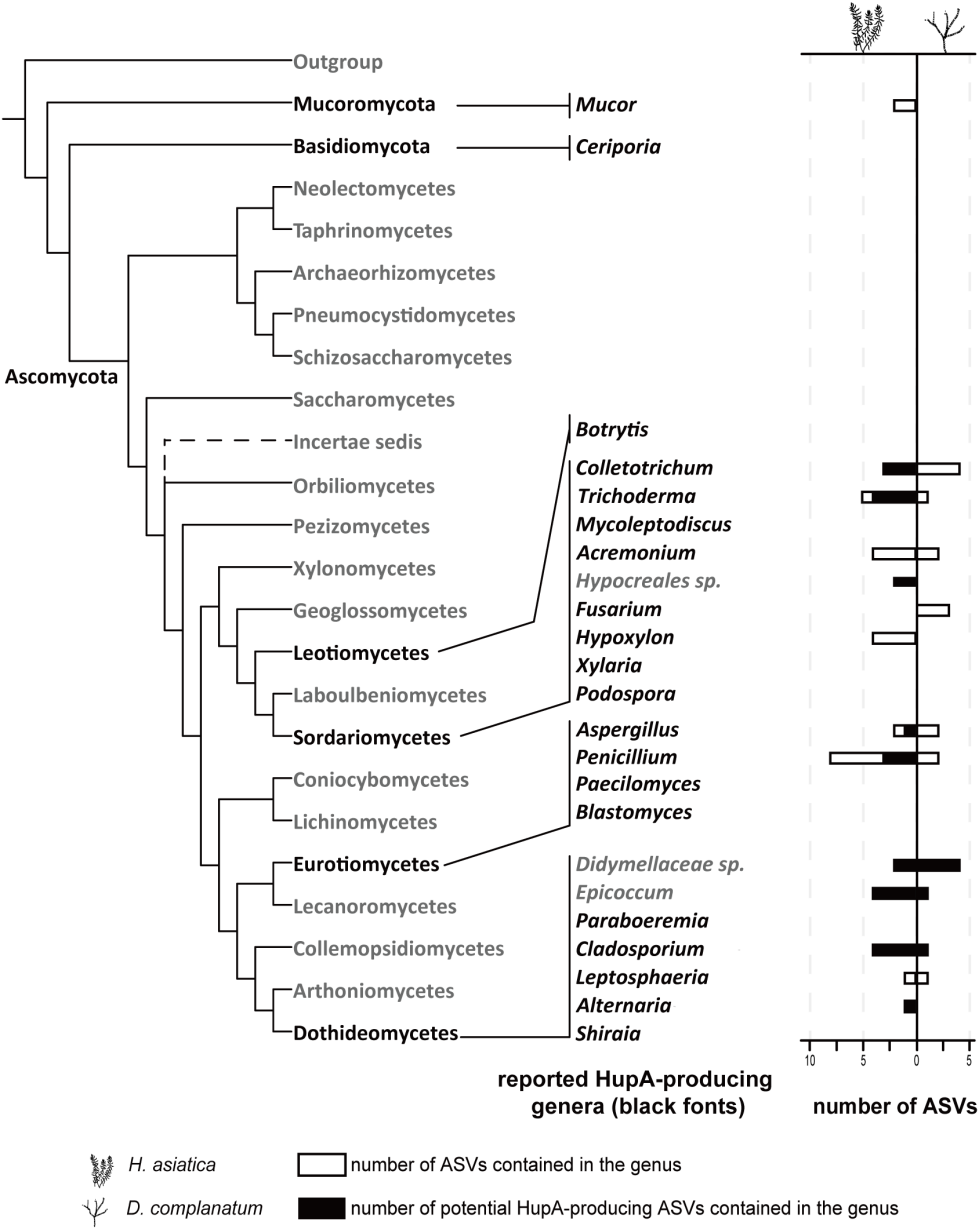
The roles of potential HupA-producing fungi in mycobiome networks. Phylogenetic distribution of the reported HupA-producing genera (black fonts). Empty horizontal bars imply the number of ASVs contained in the genus, and the horizontal bars filled with colors indicate the number of potential HupA-producing ASVs.

The absence of HupA in Lycopodioideae species was observed, but the mycobiome of *D. complanatum* contained several fungi (ASV4, ASV25, ASV38, ASV41, and ASV104) exhibiting high sequence similarity to RHP fungi. These fungi may lack HupA production ability, despite sharing high ITS sequence similarity with known HupA-producing strains. The ITS sequences used in the study represent only a small region of the fungal genome, and even minor genetic variations in other parts of the genome could lead to a loss of HupA production capacity (Kiss, 2012; Lücking et al., 2020). Alternatively, it is also possible that these fungi do possess the ability to produce HupA, but certain specific ecological factors or symbiotic associations necessary for HupA production may be lacking within *D. complanatum* (Lücking et al., 2020). As a result, the expression of genes related to HupA biosynthesis could be affected. Our network analysis provided further insights into these possibilities by examining the distinct ecological roles played by PHP fungi in the fungal interaction network of both *H. asiatica* and *D. complanatum*. According to Olesen et al. (2007), network hubs (crucial nodes throughout the network with Zi > 2.5 and Pi > 0.62), module hubs (crucial nodes within modules with Zi > 2.5 and Pi < 0.62), and connectors (connecting nodes among modules with Zi < 2.5 and Pi > 0.62) are considered as keystone microbes due to their important connecting roles. These keystone microbes also serve as intermediaries, bridging the host and abiotic factors to the plant microbial community (Agler et al., 2016). Our results suggest that almost all of the PHP fungal ASVs (4 out of 5) were peripherals (non-critical nodes with Zi < 2.5 and Pi < 0.62) in the network of *D. complanatum*. In contrast, almost half of the PHP ASVs (6 out of 13) occupied key positions in the network of *H. asiatica* as module hubs and connectors (**Figure 5**). Through complex interactions among microorganisms, they may play a vital role in system-level coordination for HupA production and overall fungal community dynamics. Furthermore, the notable interactions between PHP fungi and other fungi within the network or the same module indicated new clues for searching potential fungal facilitators. The common method to find potential fungal facilitators is to combine fungi that have similar ecological niches, since they colonize in close physical distance and embedded in one complex microbial community (Soliman and Raizada, 2013). Therefore, fungal ASVs in the same module as PHP fungal ASV may serve as fungal facilitators, mitigating the decline in HupA-producing capacity through coculture. By examining the distribution of these fungal genera across different networks, we can make a more informed assessment of their potential as facilitators. For instance, ASVs of the genera *Penicillium* and *Trichoderma* were consistently detected in more than four modules containing PHP fungi in both *H. asiatica* networks (**Supplementary Figure S8**), making them the most promising candidates for future co-cultivation investigations. Additionally, ASVs of the genera *Dioszegia*, *Exobasidium*, *Lycoperdon*, and *Cladosporium* were also recommended as they were detected in four modules containing PHP fungi in at least one of the *H. asiatica* networks. On the contrary, caution should be exercised with fungal genera or families such as *Phaeosphaeria* and Didymellaceae, as they were predominantly associated with modules containing PHP fungi in the *D. complanatum* network, suggesting they may not be suitable facilitators. It is important to acknowledge that our experimental approach did not include culture-based techniques, which limits our ability to establish direct biosynthetic connections between specific fungi and HupA production. Future studies involving cocultures of potential HupA-producing fungi and their putative facilitators will be crucial to validate the significance of their interactions and their impact on sustainable HupA production.

Future research could expand upon our findings by incorporating species such as *Lycopodiella inundata* from the Lycopodioideae subfamily, which has been suggested as a potential source of HupA (Szypuła and Pietrosiuk, 2023). While resource constraints precluded its inclusion in our study, investigating *L. inundata* could provide valuable insights into HupA production across the Lycopodiaceae family. Furthermore, expanding the investigation of the mycobiome to Lycopodiaceae roots could provide crucial insights into the ecological and biochemical mechanisms underlying HupA production, further connecting plant–fungal symbioses of root-associated fungi with secondary metabolite biosynthesis. As the current study utilized plant samples from one location per species, to further generalize the findings and increase the robustness of the results, future studies should consider including samples of the same species from multiple locations and across broader time frames. As spatial and temporal differences in fungal community compositions and structures have been reported in the HupA-producing plant *H. serrata* (Pang et al., 2022; Shen et al., 2022), a broader sampling approach that accounts for such variation would strengthen the study’s findings and conclusions.

## 5 Conclusion

This study advances our knowledge of phyllosphere fungal diversity in Lycopodiaceae species and provides valuable insights into the search for potential HupA-producing fungi and fungal facilitators. It highlights the notable variations of HupA concentration and mycobiome assemblies across tissues, pressing the importance of exploring young tissues. Furthermore, we revealed that the potential HupA-producing fungi are often hubs in the mycobiome networks of HupA-producing plants, implying that the ecological interactions among microbes may play roles in mitigating and sustaining the fungi-mediated production of complex bioactive compounds. Future investigations into these intricate interactions will deepen our understanding of the molecular and ecological factors influencing HupA biosynthesis and its industrial application.

## Data Availability

The short read data are available in the Short Read Archive (SRA) of NCBI GenBank (BioProject: PRJNA981640). Code for sequence processing and statistical analysis is presented at GitHub (https://github.com/koshroom/Huperzia_mycobiome).
