# Supplementary material for: Phyllosphere mycobiome in two Lycopodiaceae plant species: unraveling potential HupA-producing fungi and fungal interactions
Source: Front Plant Sci. 2025 Mar 14;16:1426540. doi: 10.3389/fpls.2025.1426540 (PMC11949897; doi:10.3389/fpls.2025.1426540)
Supplement: Supplementary file 1 [file DataSheet1.docx]

Supplementary Figures

**
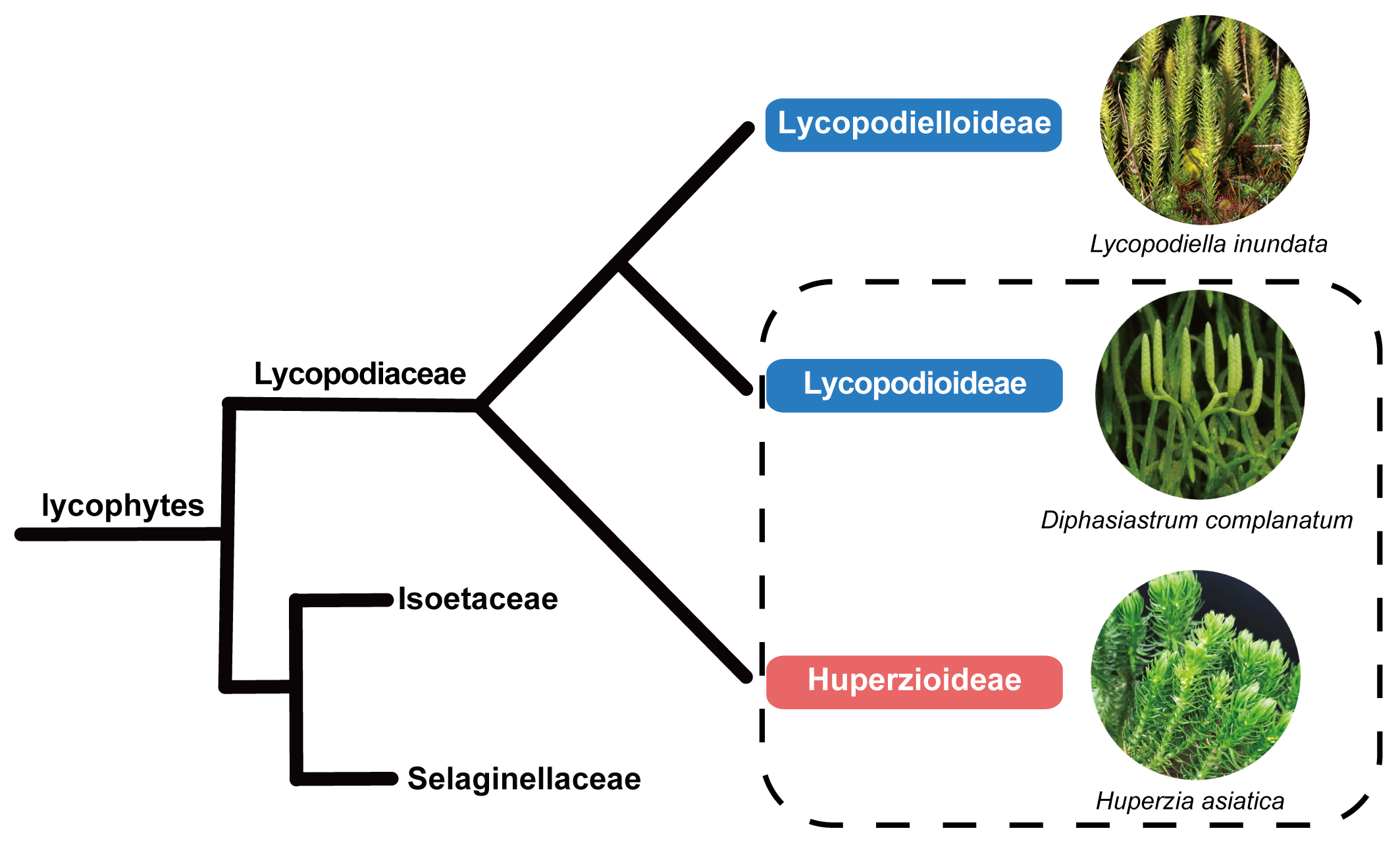
**

**Supplementary Fig. 1** Phylogeny of lycophytes. The blue-shaded subgroups do not produce HupA, and the red-shaded one can produce HupA. The dashed lines frame the materials involved in our study. Image of *D. complanatum* courtesy of P.-F. Lu. Image of *L. inundata* courtesy of Wikipedia.


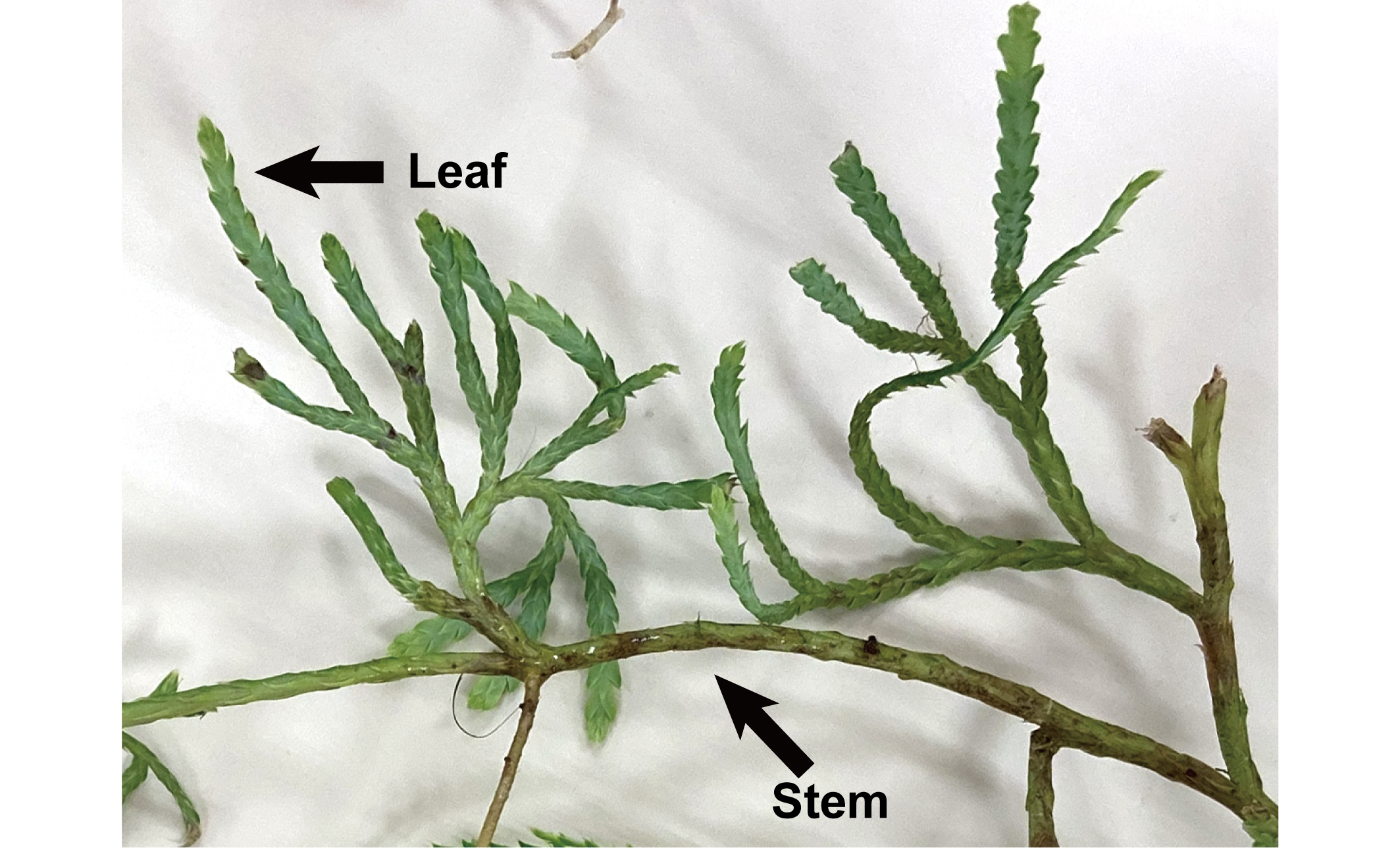


**Supplementary Fig.2** Stem and leaf of *Diphasiastrum complanatum*.


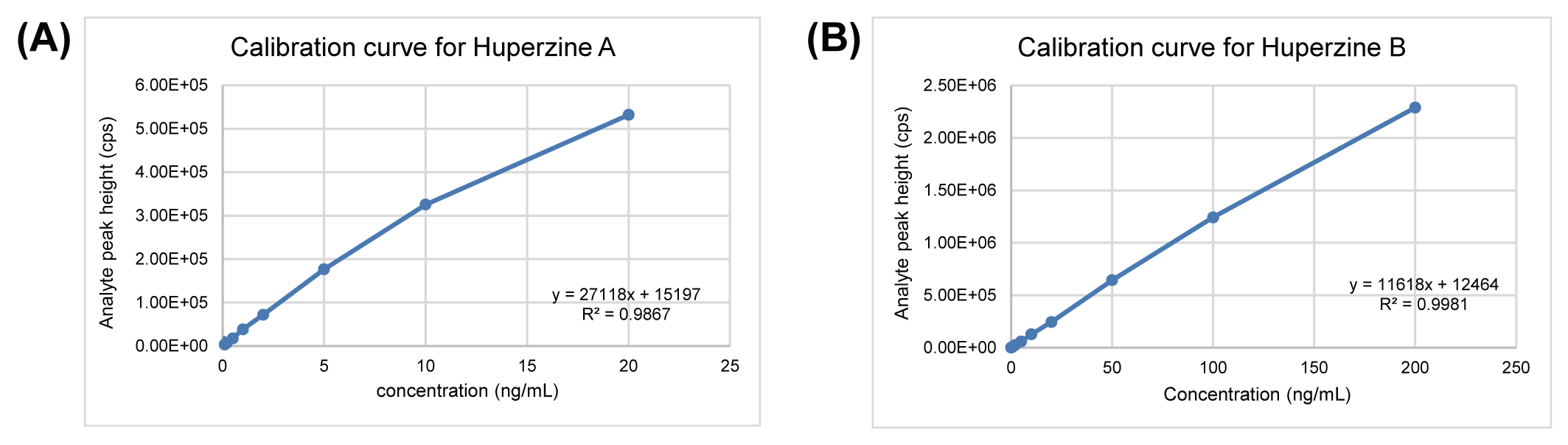


**Supplementary Fig.3** LC-MS calibration curves for Huperzine A and Huperzine B.

**
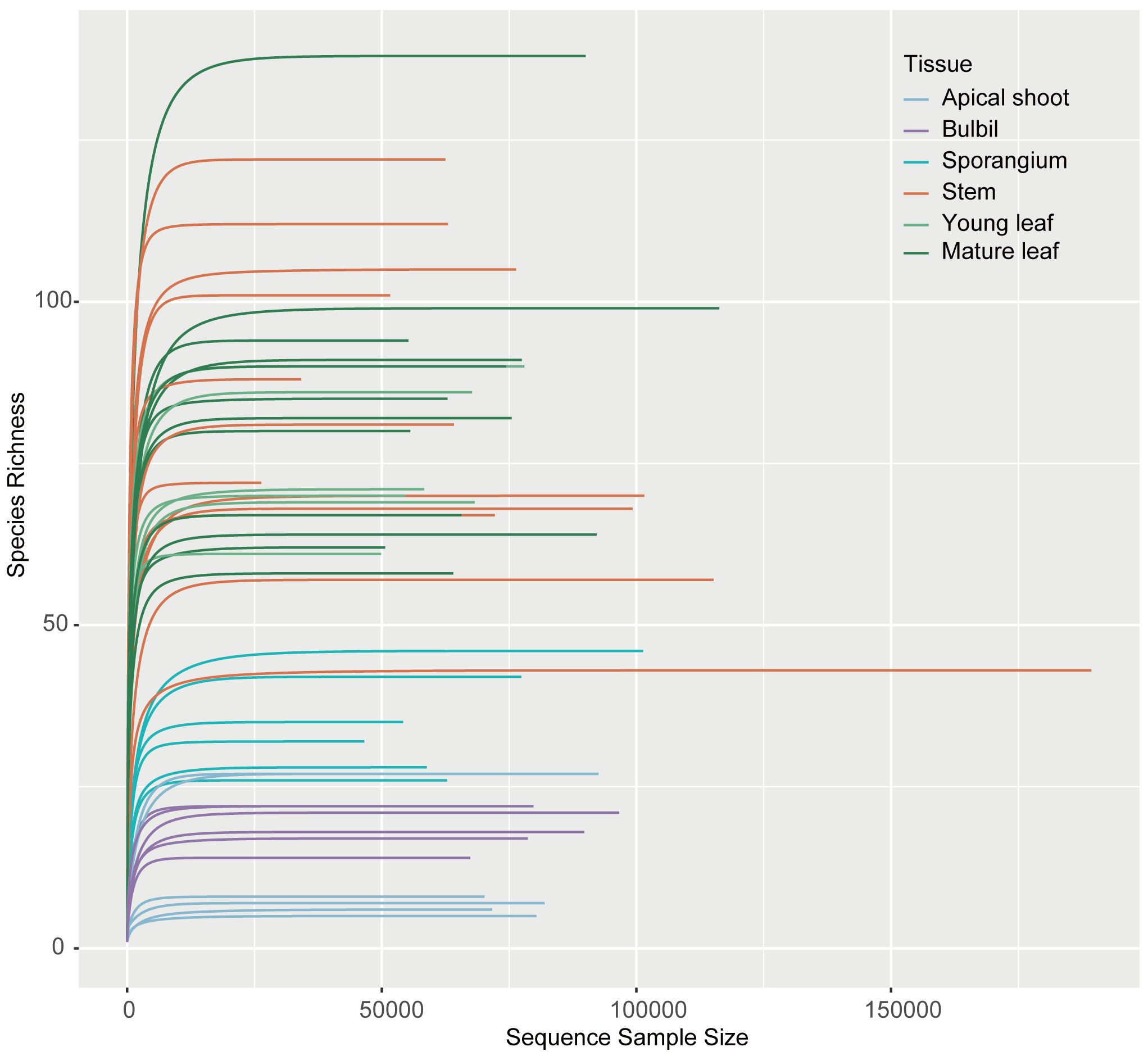
**

**Supplementary Fig. 4** The species accumulation curves.


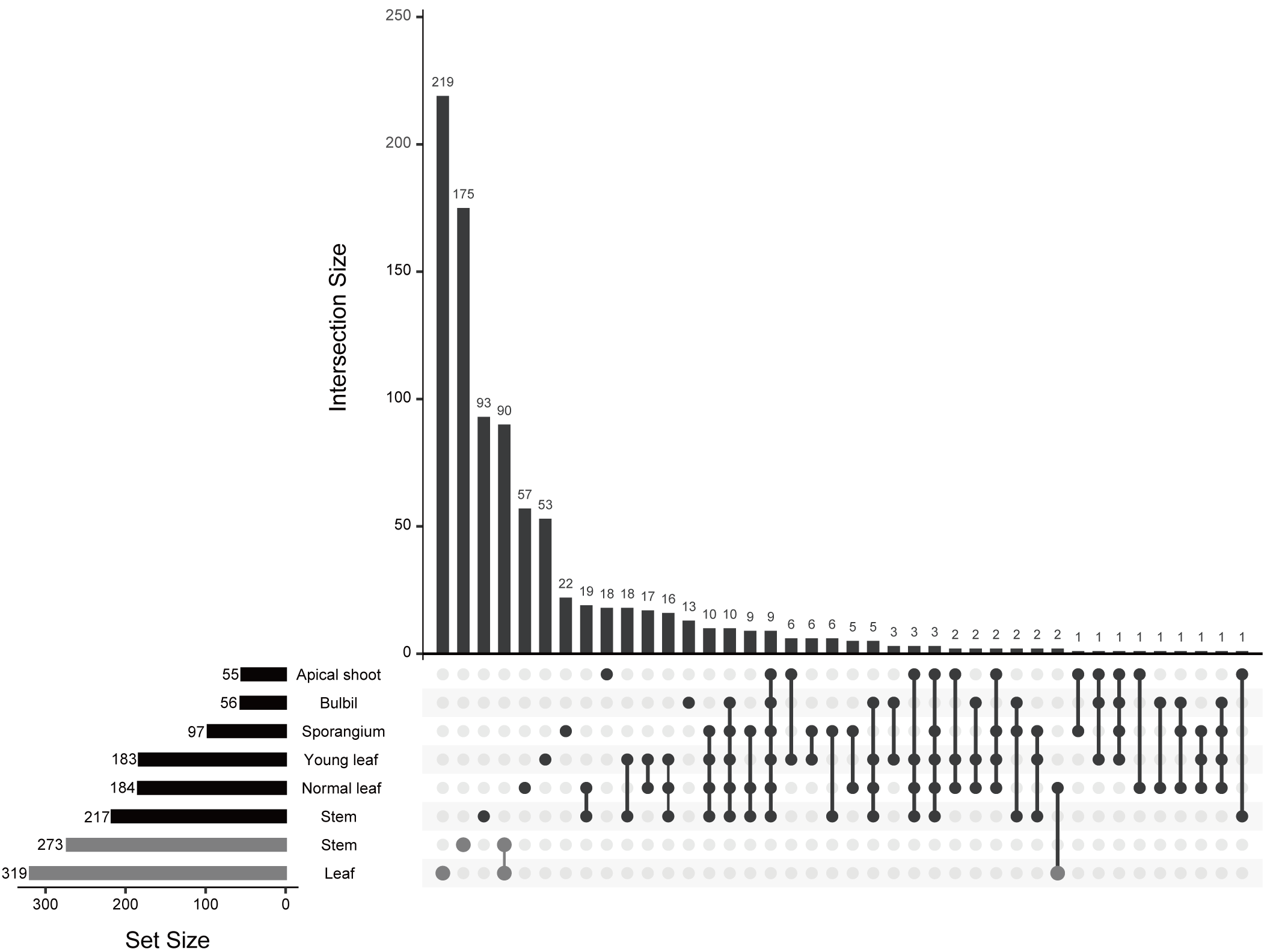


**Supplementary Fig. 5** UpSet plot showing the unique and intersected ASVs among samples. Total ASVs in each sample are displayed through set size, in which black bars are sets of *Huperzia asiatica* and gray bars are sets of *Diphasiastrum complanatum*. The vertical bars represent the distinct and overlapping ASVs in different tissues, and dots below indicate the samples included in each vertical bar.


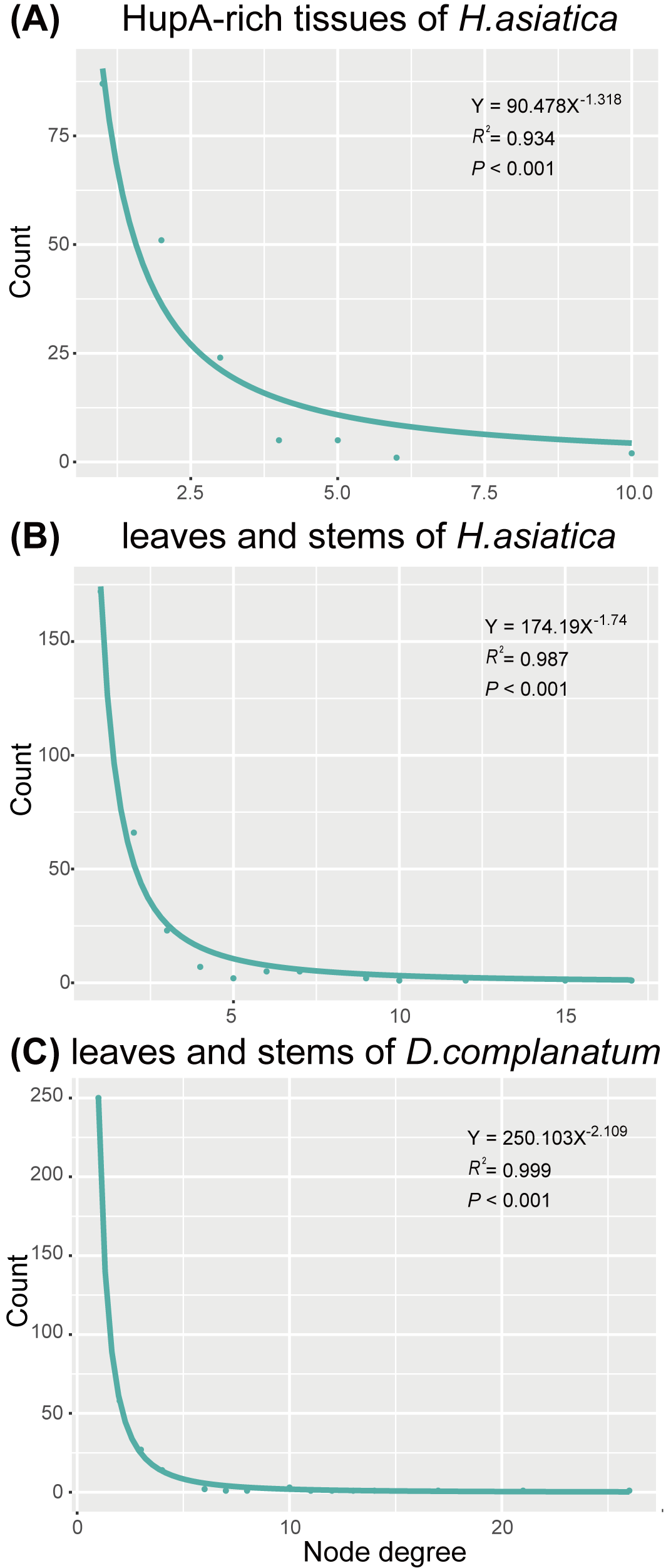


**Supplementary Fig. 6** The distribution curves of network node degrees fit with the power-law model.


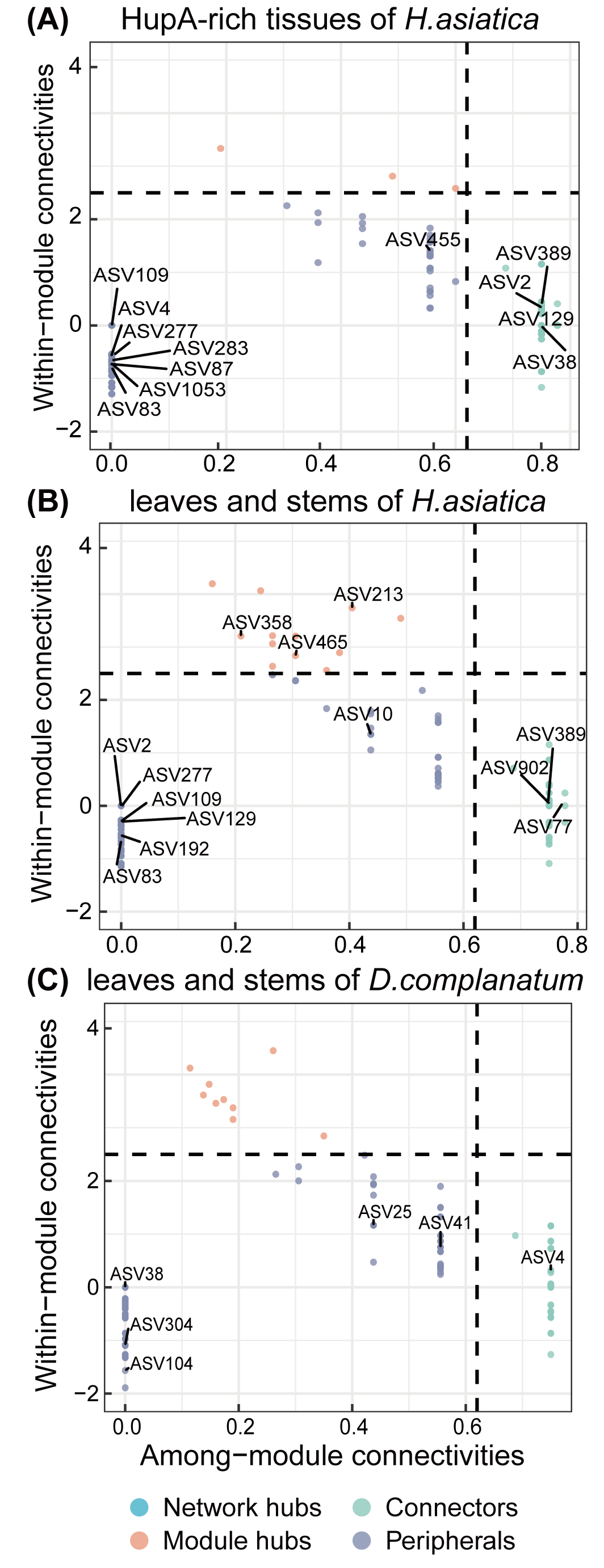


**Supplementary Fig. 7** Intra-module connectivity (Zi) and inter-module connectivity (Pi) plots.


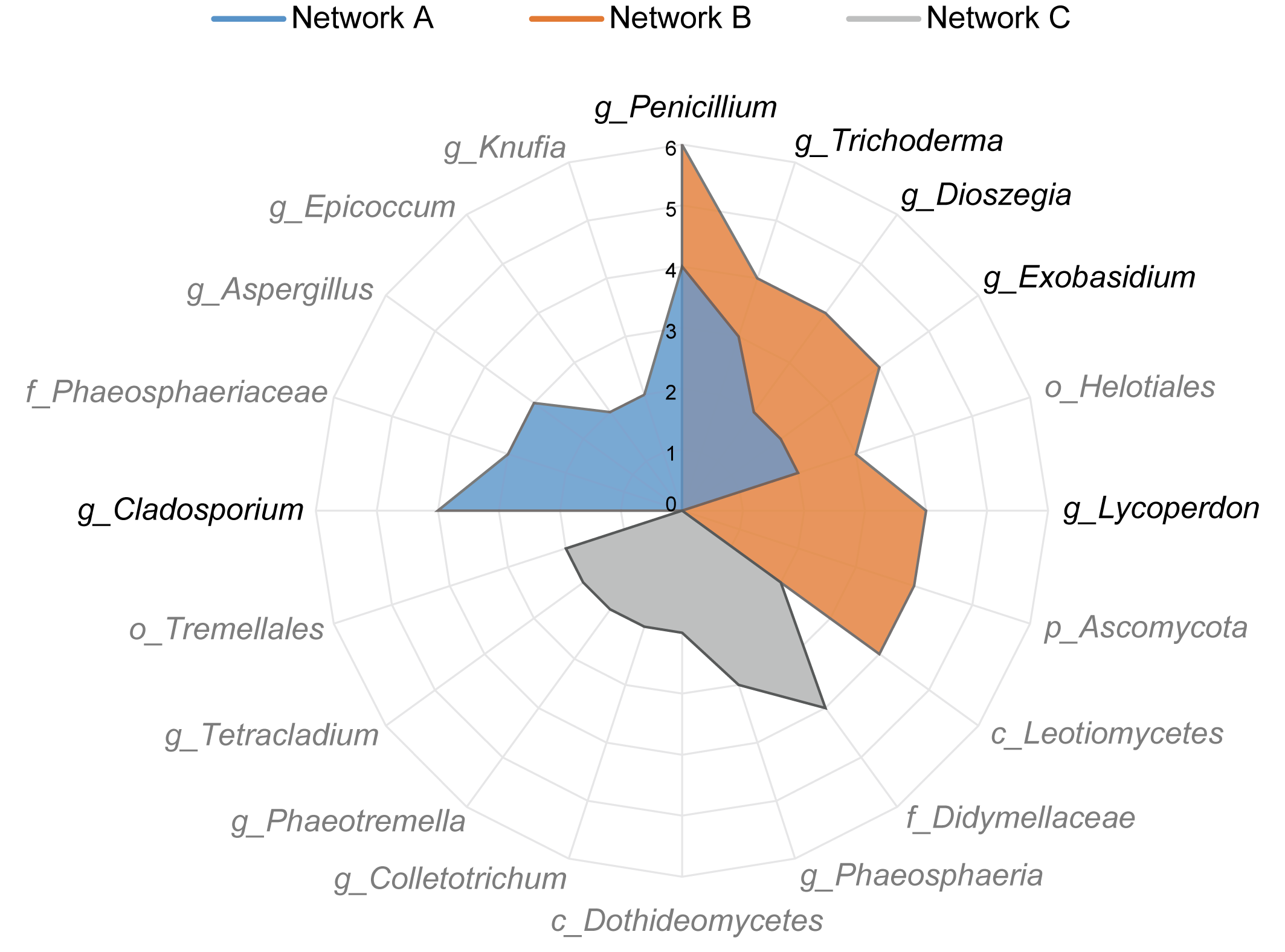


**Supplementary Fig. 8** A radar plot comparing the number of ASVs belong to the main fungal genera within modules containing PHP fungi in network A (HupA-rich tissues of *Huperzia asiatica*, i.e., bulbils, apical shoots, and young leaves), network B (leaves and stems of H. asiatica) and Network C (leaves and stems of *Diphasiastrum complanatum*). The black font labels candidates that we believe are promising for future co-cultivation investigations (i.e. was detected in at least four modules in Network A or B).
